# Voluntary Exercise to Reduce Anxiety Behaviour in Traumatic Brain Injury Shown to Alleviate Inflammatory Brain Response in Mice

**DOI:** 10.3390/ijms24076365

**Published:** 2023-03-28

**Authors:** Xiaoxuan Hu, Yuhang Ou, Jiashuo Li, Meiqi Sun, Qian Ge, Yongqi Pan, Zhenlu Cai, Ruolan Tan, Wenyu Wang, Jing An, Haixia Lu

**Affiliations:** 1 Department/Institute of Neurobiology, School of Basic Medical Sciences, Xi’an Jiaotong University Health Science Center, Xi’an 710061, China; 2 Key Laboratory of Ministry of Education for Environment and Genes Related to Diseases, Xi’an Jiaotong University Health Science Center, Xi’an 710061, China; 3 Department of Human Anatomy & Histoembryology, School of Basic Medical Sciences, Xi’an Jiaotong University Health Science Center, Xi’an 710061, China

**Keywords:** traumatic brain injury, voluntary exercise, anxiety behavior, neuroinflammatory response, NLRP3 inflammasome

## Abstract

Traumatic brain injury is a leading cause of neuroinflammation and anxiety disorders in young adults. Immune-targeted therapies have garnered attention for the amelioration of TBI-induced anxiety. A previous study has indicated that voluntary exercise intervention following TBI could reduce neuroinflammation. It is essential to determine the effects of voluntary exercise after TBI on anxiety via inhibiting neuroinflammatory response. Mice were randomly divided into four groups (sham, TBI, sham + voluntary wheel running (VWR), and TBI + VWR). One-week VWR was carried out on the 2nd day after trauma. The neurofunction of TBI mice was assessed. Following VWR, anxiety behavior was evaluated, and neuroinflammatory responses in the perilesional cortex were investigated. Results showed that after one week of VWR, neurofunctional recovery was enhanced, while the anxiety behavior of TBI mice was significantly alleviated. The level of pro-inflammatory factors decreased, and the level of anti-inflammatory factors elevated. Activation of nucleotide oligomerization domain-like thermal receptor protein domain associated protein 3 (NLRP3) inflammasome was inhibited significantly. All these alterations were consistent with reduced microglial activation at the perilesional site and positively correlated with the amelioration of anxiety behavior. This suggested that timely rehabilitative exercise could be a useful therapeutic strategy for anxiety resulting from TBI by targeting neuroinflammation.

## 1. Introduction

Traumatic brain injury (TBI) is a major health hazard that is accompanied by high rates of mortality and disability [1]. Approximately 20 million individuals worldwide are affected by TBI, and approximately 2.5–6.5 million suffer from the complications of TBI [2,3,4]. In the last few decades, mental and psychological disorders, especially anxiety, have received considerable attention during trauma treatment and rehabilitation [3,5]. According to the Centers for Disease Control and Prevention, approximately 3–28% of patients with TBI in the USA suffer from anxiety, and the outcomes are less than ideal [5]. Anxiety is the second most common neuropsychiatric disorder in patients with persistent neuropsychiatric symptoms after TBI [6,7,8]. Meanwhile, anxiety behavior after TBI was variable and exhibited a time-dependent increase for up to 3 weeks [6,9,10,11,12]. Further exploration of appropriate intervention measures for anxiety after TBI is warranted to refine treatment guidelines.

Neuroinflammation is a central pathophysiological feature of TBI. Immediately after TBI, an acute immune cascade was initiated, and then cellular inflammatory levels reached a peak within seven days [13,14]. Glia cells, including astrocytes and microglia, have been activated, which showed in typical morphology, and secreting of various chemokines, cytokines and factors were enhanced [15,16]. These cells are considered the key player in initiating neuroinflammatory responses [16]. The nucleotide oligomerization domain-like receptor thermal protein domain associated protein 3 (NLRP3) in microglia is involved in the neuroinflammatory response to brain tissue damage. Studies have confirmed that the activation of the NLRP3 inflammasome and the release of pro-inflammatory cytokines were greatly up-regulated following TBI [17,18,19,20]. Consequently, the NLRP3 inflammasome is considered to be a potential therapeutic target for the management of neuroinflammation after the TBI [21]. Previous studies have demonstrated that cortical neuroinflammation is associated with behavioral deficits following TBI [22]. Additionally, secondary injury, characterized by gliosis, plays an important role in the post-TBI mood disorders [23]. Recent research has indicated that suppressing the ongoing inflammatory response after TBI could be the key to unlocking the treatment of post-trauma anxiety [24,25,26]. Evidence from rodent studies has confirmed that the suppression of NLRP3 inflammasome activation can produce dramatic improvement in neurofunctional outcomes following TBI with respect to the molecular biological and pharmacological aspects [18,27,28,29,30,31].

Voluntary exercise has been proven to attenuate neuronal loss, inhibit glia activation, reduce neuroinflammation, improve mood, and subsequently facilitate recovery after TBI [32,33,34,35]. Existing data showed that voluntary exercise or voluntary exercise pre-conditioning facilitated neuroplasticity after TBI via the reduction in the level of inflammatory factors and elevation in the level of brain-derived neurotrophic factor [36,37,38,39,40]. However, other studies showed contradictory effects, depending on the different exercise strategy [36,37,38,41]. Furthermore, since the heightened stress response occurred within the first two weeks after TBI, the ideal time of voluntary exercise after TBI remains to be determined [36]. In addition, the possible relationship between the alleviation of injury induced-anxiety and inhibition of inflammatory response based on glia cell activation after voluntary exercise remains to be further analyzed. In the current study, the mice carried out a one-week voluntary wheel running (VWR) which started on the 2nd day after TBI. Thereafter, neurofunctional recovery, anxiety behaviors and neuroinflammatory response were investigated.

## 2. Results

### 2.1. VWR Improved the Neurological Recovery of TBI Mice

**Figure 1:**
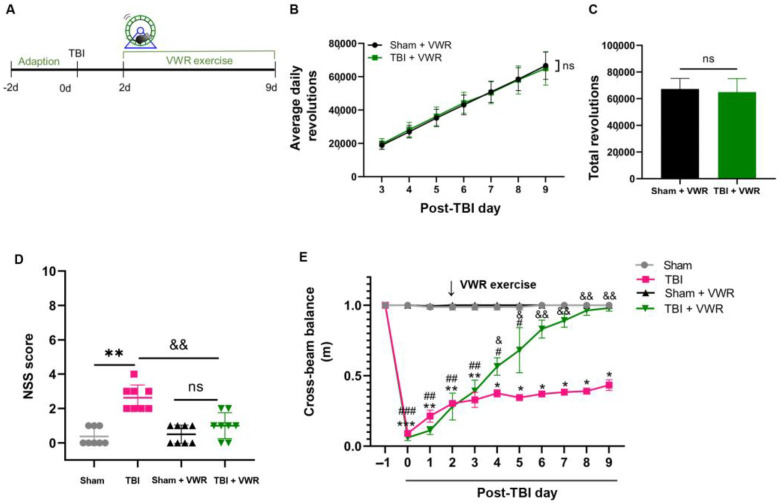
VWR improved sensorimotor coordination and balance in TBI mice. (**A**) The diagrammatic sketch of experimental design and VWR strategy. (**B**) There was no significant difference in daily running wheel revolutions between TBI and Sham groups. Two-way ANOVA, post hoc Tukey’s multiple comparisons tests; *n* = 7. (**C**) No significant difference was found in total running wheel revolutions between TBI and Sham groups. Unpaired student’s t-test; *n* = 7. (**D**) TBI-induced elevation of NSS significantly reduced after seven days of VWR. One-way ANOVA, post hoc Sidak’s multiple comparisons tests; *n* = 12. (**E**) TBI caused a significant reduction in beam walking distance increased gradually during VWR. Two-way ANOVA, post hoc Tukey’s multiple comparisons tests; *n* = 6). *** *p* < 0.0001, ** *p* < 0.001, * *p* < 0.01, vs. Sham. ^&^ *p* < 0.01, ^&&^ *p* < 0.001 vs. TBI. ^###^ *p* < 0.0001, ^##^ *p* < 0.001, ^#^ *p* < 0.01 vs. Sham + VWR. ns, no significance. VWR, voluntary wheel running; TBI, traumatic brain injury; NSS, neurological severity score.

In this study, damage to motor coordination and brain tissue of the TBI mice was investigated. Neurological functional impairment, as detected by the neurological severity score (NSS), was observed (t = 17.16, df = 10, *p* < 0.0001; Figure S1A), accompanied by prominent tissue loss two days after TBI (Figure S1B), and surge in the number of c-fos positive neurons (Figure S1C) in the perilesional cortex 1.5 h after TBI. VWR was initiated on the 2nd day after TBI and lasted for seven days (Figure 1A). No significant difference was observed in the frequency of daily (F_(1, 12)_ = 0.0009115, *p* = 0.9764) and total (t = 0.1826, df = 12, *p* = 0.8582) running wheel revolutions between the sham and TBI groups (Figure 1B,C), suggesting that the willingness and ability to perform exercise were comparable for the mice from both groups. As anticipated, the motor coordination of the TBI mice improved significantly after VWR, evidenced by the reduction in the NSS (F_(3, 28)_ = 20.45, *p* < 0.0001; Figure 1D) and rapidly increase in the distance walked across the wooden beam (F_(3, 7)_ = 284, *p* < 0.0001; Figure 1E).

### 2.2. VWR Eased Anxiety in TBI Mice

**Figure 2:**
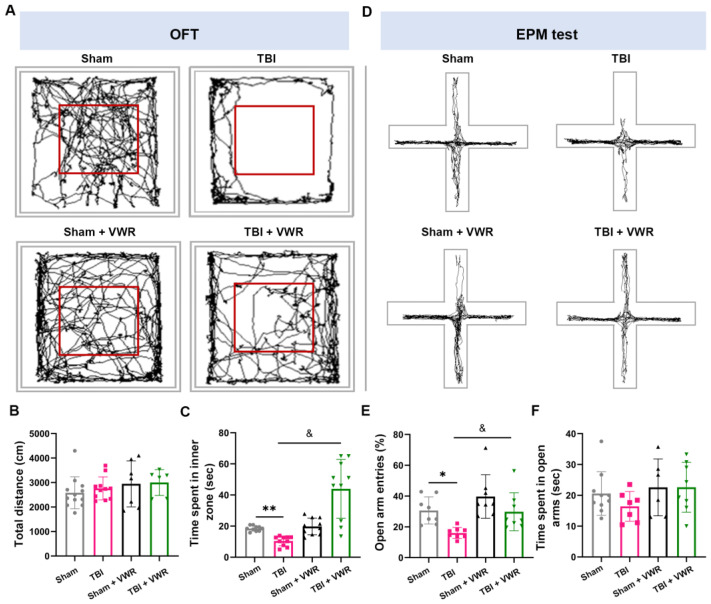
VWR eased the anxiety behavior of TBI mice. (**A**,**D**) Representative traces of locomotor activity in the OFT and EPM test. (**B**) No significant difference in total distance was observed between the four groups in the open field. (**C**) TBI significantly decreased the time that mice spent in the inner zone of the open field, while VWR significantly reversed it. (**E**) TBI significantly decreased while the VWR significantly increased the open-arm entries of mice. (**F**) The total times spent in open arms were similar between the four groups. One-way ANOVA, Post hoc Sidak’s multiple comparisons tests; *n* = 8. ** *p* < 0.001, * *p* < 0.05 vs. Sham, ^&^ *p* < 0.05 vs. TBI. VWR, voluntary wheel running; TBI, traumatic brain injury; OFT, open field test; EPM, elevated plus maze.

Locomotor activity in the open field test (OFT) and elevated plus maze (EPM) test was assessed after seven days of exercise to evaluate the anxiety behavior of the mice. The total distance in the OFT did not differ among the four groups (F_(3, 38)_ = 1.115, *p* = 0.355; Figure 2A,B). Notably, the TBI mice spent significantly lesser time in the central area compared to the mice in the sham group. Nevertheless, after one week of exercise, the mice spent a substantially longer time in the central area, which was almost identical to that in the sham group (F_(3, 36)_ = 10.40, *p* < 0.0001; Figure 2A,C). In the EPM test, the number of entries in the open arms was low in the TBI group, while this phenomenon was completely reversed after one week of VWR (F_(3, 36)_ = 20.99, *p* < 0.0001; Figure 2D,E). No difference was observed in the time spent in the open arms among the different groups (F_(3, 28)_ = 1.876, *p* = 0.1566; Figure 2D,F).

### 2.3. VWR Inhibited the Neuroinflammation Response after TBI

**Figure 3:**
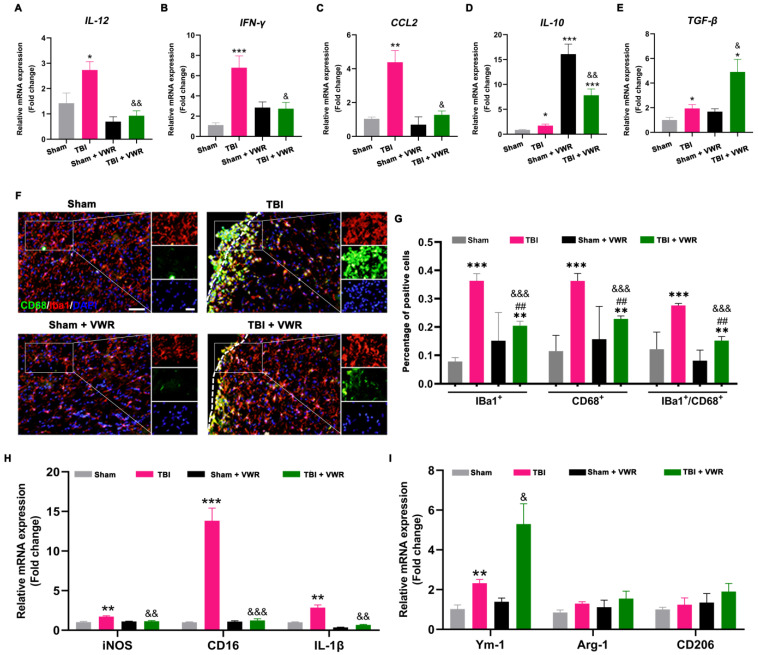
VWR reduced the inflammatory response after TBI. (**A**–**C**) The mRNA expression of pro-inflammatory factors in the perilesional cortex, including IL-12, IFN-γ, and CCL2, significantly increased after TBI and decreased after VWR. (**D**,**E**) The mRNA expression of anti-inflammatory factors, including IL-10 and TGF-β, increased after TBI and VWR. However, the VWR-induced increase of IL-10 was independent of TBI. (**F**,**G**) The activation of microglia in the perilesional cortex was noticed after TBI and significantly reduced after VWR, showed by the percentage of Iba1^+^, CD68^+^ and Iba1^+^/CD68^+^ cells. (**H**) The mRNA expression of iNOS, CD16, and IL-1β (M1 microglia markers) in the perilesional cortex significantly increased after TBI and decreased after VWR. (**I**) The mRNA expression of Ym-1 (M2 microglia markers) significantly increased after TBI and was further enhanced after VWR. While the expression of Arg-1 and CD206 (M2 microglia markers) slightly increased after TBI but significantly up-regulated in TBI + VWR mice. *** *p* < 0.0001, ** *p* < 0.001, * *p* < 0.05 vs. Sham. ^&&&^ *p* < 0.0001, ^&&^ *p* < 0.001, ^&^ *p* < 0.05 vs. TBI. ^##^ *p* < 0.001 vs. Sham + VWR. One-way ANOVA, Post hoc Sidak’s multiple comparisons tests; *n* = 4. Iba1: red; CD68: green; DAPI: blue; scale bar = 100 µm. VWR, voluntary wheel running; TBI, traumatic brain injury; IL-12, interleukin 12; IFN-γ, interferon γ; CCL2, the C-C motif chemokine ligand 2; IL-10, interleukin 10; TGF-β, transforming growth factor-β; Iba1, Ionized calcium-binding adaptor molecule 1.

To evaluate the effect of VWR on inflammation reaction following TBI, the mRNA expression levels of three pro-inflammatory factors and two anti-inflammatory factors were detected using quantitative real-time polymerase chain reaction (qRT-PCR) on day nine after TBI. After VWR for seven days, TBI-induced inflammation declined significantly, represented by the reduction of pro-inflammatory factors, including interleukin (IL)-12 (F_(3, 29)_ = 10.82, *p* < 0.0001), interferon (INF)-γ (F_(3, 31)_ = 11.86, *p* < 0.0001) and chemokine ligand (CCL) 2 (F_(3, 30)_ = 18.07, *p* < 0.0001; Figure 3A–C). Concurrent with this finding, the anti-inflammatory effect of VWR was observed, represented by the marked elevation in IL-10 (F_(3, 28)_ = 31.50, *p* < 0.0001) and transforming growth factor (TGF)-β (F_(3, 23)_ = 10.62, *p* = 0.0001; Figure 3D,E). It is noteworthy that the level of IL-10 rose dramatically after VWR, irrespective of the presence or absence of TBI. The degree of VWR-induced elevation in IL-10 after TBI was significantly lower than that in the sham group (Figure 3D). This indicated that IL-10 and TGF-β might act under a different mechanism (Figure 3E).

### 2.4. VWR Attenuated the Activation of Microglia and Promoted M2 Phenotypic Polarization after TBI

Microglia plays a critical role in neuroinflammatory responses that further contribute to secondary injury after TBI. To evaluate the effect of VWR on the activation and polarization of microglia, the number of activated microglia (Ionized calcium-binding adaptor molecule 1 (Iba1)^+^/CD68^+^ cells) in the perilesional cortex was counted after immunofluorescence staining, the mRNA expression of M1 microglia markers (iNOS, CD16, IL-1β) and M2 microglia markers (Ym-1, Arg-1, CD206) was detected by qRT-PCR on day nine after TBI. Results showed that the alteration of inflammatory factors was accompanied by the activation of microglia (Figure 3F–I). The density of activated microglia in TBI mice was higher than that in the other three groups, presented by the increase in the number of Iba1^+^ (F_(3, 12)_ = 5.305, *p* = 0.0147), CD68^+^ (F_(3, 28)_ = 9.333, *p* = 0002), and Iba1^+^/ CD68^+^ (F_(3, 12)_ = 5.354, *p* = 0.0143) cells in the perilesional somatosensory cortex (SSC). It significantly decreased after VWR (Figure 3F,G). Changes in the number of Iba1^+^/ CD68^+^ (F_(3, 24)_ = 47.03, *p* = 0.0001) cells in the corpus callosum (CC) were consistent with SSC (Figure S2). However, there were no significant differences in the number of Iba1^+^/ CD68^+^ cells in the contralateral cortex (Figure S3). Meanwhile, obviously an increase in the mRNA expression of M1 microglia markers (iNOS (F_(3, 18)_ = 9.959, *p* = 0.0004), CD16 (F_(3, 15)_ = 79.80, *p* < 0.0001), IL-1β (F_(3, 22)_ = 36.44, *p* < 0.0001)) after TBI and decrease after VWR were found (Figure 3H). To the expression of M2 microglia markers, Ym-1 (F_(3, 10)_ = 40.78, *p* < 0.0001) significantly increased after TBI and further enhanced after VWR. While the expression of Arg-1 (F_(3, 13)_ = 4.110, *p* = 0.0296) and CD206 (F(_3, 19)_ = 6.271, *p* = 0.0039) slightly increased after TBI but significantly elevated in TBI + VWR mice (Figure 3I).

### 2.5. VWR Eased Anxiety Behavior in Mice via the Inhibition of NLRP3 Activation

**Figure 4:**
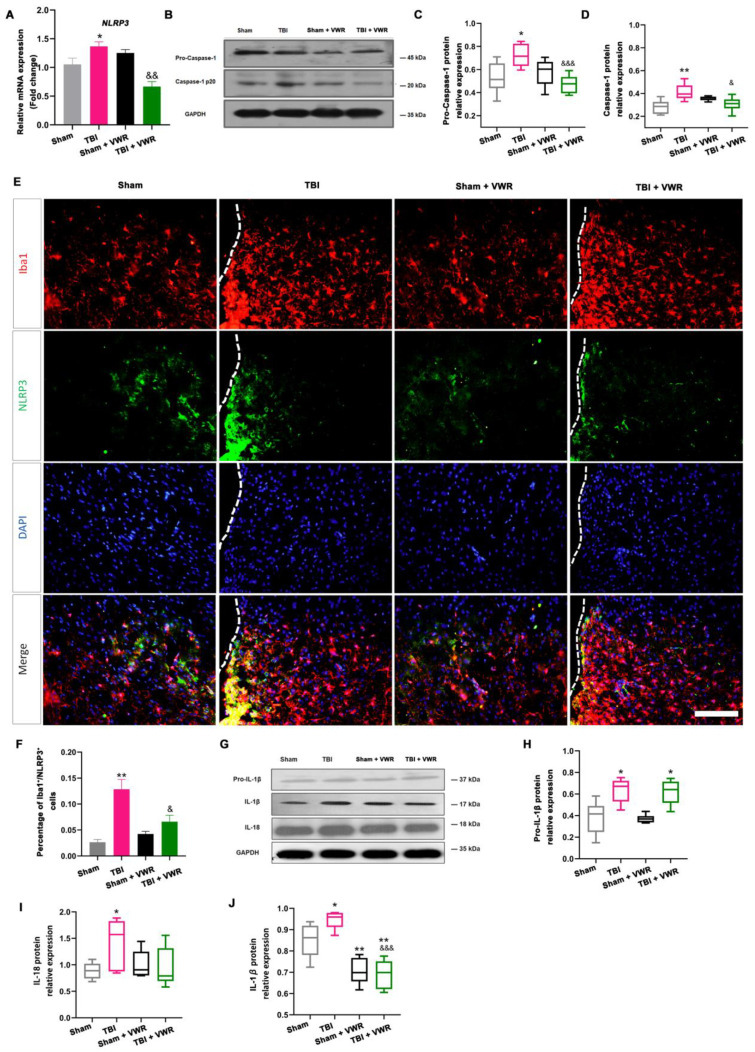
VWR reduced the activation of NLRP3 signaling. (**A**) TBI-induced elevation of NLRP3 mRNA in the perilesional cortex significantly decreased after VWR. (**B**–**D**) TBI-induced increase of pro-caspase-1 and caspase-1 proteins in the perilesional cortex significantly decreased after VWR. (**E**,**F**) Consistently, the increased expression of NLRP3 (green in **E**) in activated microglia (red in **E**) significantly decreased after VWR. (**G**,**H**) The protein level of pro-IL-1β increased significantly after TBI. However, it did not affect by VWR. (**G**,**J**) The protein level of mature IL-1β increased significantly after TBI and remarkably dropped after VWR. The VWR-induced reduction was independent of injury. (**G**,**I**) The protein level of IL-18 was similar to NLRP3 and significantly increased after TBI. ** *p* < 0.01, * *p* < 0.05 vs. Sham, ^&&&^ *p* < 0.0001, ^&&^ *p* < 0.01, ^&^ *p* < 0.05 vs. TBI. One-way ANOVA, Post hoc Sidak’s multiple comparisons tests; *n* =5. Iba1: red; NLRP3: green; DAPI: blue; scale bar = 100 µm. VWR, voluntary wheel running; NLRP3, NOD-like receptor thermal protein domain associated protein 3; TBI, traumatic brain injury; Iba1, Ionized calcium-binding adaptor molecule 1; IL-1β, interleukin 1β; IL-18, interleukin 18.

**Figure 5:**
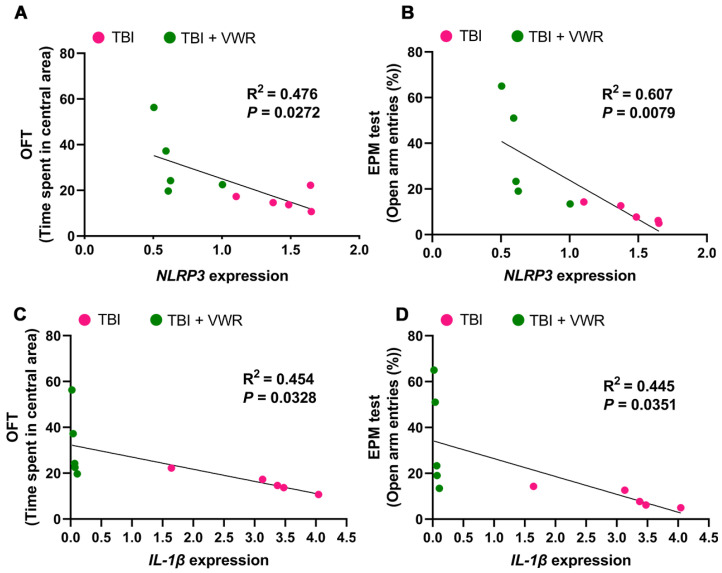
Correlation analysis between anxiety behavior and inflammasome activation. (**A**,**B**) Positive correlation between reduced NLRP3 mRNA and eased anxiety behavior in OFT and EPM test. (**C**,**D**) Positive correlation between decreased IL-1β mRNA and alleviated anxiety behavior in OFT and EPM test. Two-tailed Mann–Whitney U-test, *n* = 5. NLRP3, NOD-like thermal receptor protein domain associated protein 3; IL-1β, interleukin 1β; OFT, open field test; EPM, elevated plus maze.

NLRP3 inflammasome activation and its correlation with anxiety behavior were analyzed to further investigate the possible anti-anxiety mechanism of VWR following TBI. Two important inflammatory mediators, NLRP3 and pro-caspase-1, were detected on day nine after TBI. The expression of NLRP3 mRNA (F_(3, 32)_ = 17.17, *p* < 0.0001; Figure 4A) and pro-caspase-1 (F_(3, 28)_ = 8.266, *p* = 0.0004; Figure 4B,C) were significantly elevated after TBI and reduced by VWR. This led to the synchronous alteration of the NLRP3 inflammasome (F_(3, 28)_ = 33.28, *p* < 0.0001; Figure 4E,F)) and mature caspase-1 (F_(3, 24)_ = 6.349, *p* = 0.0002; Figure 4B,D) in the microglia. Moreover, pro-IL-1β, IL-1β and IL-18 were also detected. The expression of pro-IL-1β was significantly elevated after TBI but was not affected by VWR (F_(3, 20)_ = 10.16, *p* = 0.0003; Figure 4G,H). However, the expression of mature IL-1β showed a different alteration pattern. It rose significantly after TBI and plummeted remarkably after VWR; the VWR-induced reduction was independent of brain injury (F_(3, 20)_ = 18.34, *p* < 0.0001; Figure 4G,J). A similar tendency was noted for the alteration in IL-18 with NLRP3 (F_(3, 28)_ = 4.982, *p* = 0.0068; Figure 4G,I). Further analysis revealed a positive correlation between the reduction of NLRP3/IL-1β mRNA and amelioration in anxiety in the OFT (Figure 5A,C) and EPM tests (Figure 5B,D).

## 3. Discussion

The increased risk of psychiatric disorders after TBI has received tremendous attention during the past decade [42]. Both clinical cases and epidemiological investigations supported that anxiety was more prevalent in patients with a TBI [42,43,44,45]. In the current study, neurological functional deficits, including motor coordination impairment and anxiety behavior, were clearly observed immediately after TBI and were accompanied by marked brain tissue damage. The mice spent significantly less time in the central area of the OF, with fewer entries into the open arms in the EPM, indicating avoidance behavior. VWR was performed for one week, commencing on the 2nd day after TBI. We found that the anxiety behavior of mice was significantly alleviated and accompanied by a dramatic reduction in trauma-induced neuroinflammation. The recovery of neurological function in the TBI mice was enhanced, while NLRP3 activation was reduced.

Previous studies have shown that anxiety behavior after TBI increased in a time-dependent manner [9,10]. The stress response is heightened during the first two weeks of post-injury [36]. Therefore, timely intervention soon after injury may be extremely critical to alleviating anxiety behavior. In the current study, VWR was performed on the 2nd day after TBI since voluntary physical activity and exercise are considered to be the essential rehabilitative interventions following brain injury [33,34]. We observed that the total running distance and the total number of wheel rotations executed by the TBI mice, which indicated the willingness and ability to exercise, were comparable with those of the control mice within the period of seven days. After seven days of VWR, a reduction in anxiety was achieved successfully, in addition to the improvement in motor coordination. Although some previous studies have demonstrated that delay in instituting voluntary exercise after TBI facilitates neuroplasticity [36,37,38], other experiments have shown that exercise improved rat memory irrespective of the temporal schedule, i.e., with immediate and delayed implementation after injury [37]. Our results further confirmed that it is possible to employ voluntary exercise at the early stage (2 days after TBI) to ease anxiety behavior in TBI mice.

Neuroinflammation is one of the typical responses after brain injury. Cellular inflammatory signaling, including pro-inflammatory and anti-inflammatory signaling, is initiated immediately after TBI, which typically peaks within seven days of injury [46]. Immune-targeted therapies have been fully considered for the treatment of anxiety and mood disorders based on the relationship between inflammation and the neuropsychiatric risk after the TBI [47,48]. Evidence shows that the reversal of inflammation, whether at the acute or delayed stage, could efficiently ameliorate the development and maintenance of anxiety behavior after the TBI [25,26,49]. Consistent with these findings [50,51], we found a surge in both pro-inflammatory and anti-inflammatory signals after TBI, albeit to different degrees. The anti-inflammatory markers, especially IL-10, were markedly elevated after VWR. The alteration in these cellular signals was consistent with microglial activation at the perilesional site. It was obviously noted that M1 microglia was further reduced while the M2 microglia polarization was promoted after VWR in TBI mice. This finding demonstrated that initiating VWR at an early stage could efficiently ameliorate injury-induced neuroinflammation.

In accordance with the above-mentioned reduction in neuroinflammation, we noticed that NLRP3 inflammasome activation was inhibited by VWR. The expression of NLRP3 in the microglia, both caspase-1 activation and proinflammatory cytokines IL-1β/IL-18 secretion were also significantly inhibited. This VWR-induced inhibition was positively correlated with the alleviation of anxiety behavior in TBI mice. Although the underlining mechanisms were not clear, the increasing evidence has indicated that inhibiting inflammatory responses could relieve anxiety emotion [21].

## 4. Materials and Methods

### 4.1. Animals

Fifty-four male Kunming mice (8 weeks old, weight: 28–32 g) were obtained from the Medical Animal Center of Xi’an Jiaotong University and housed in a humidity- and temperature-controlled (40–60%, 20–24 °C) environment under a 12-h/12-h light/dark cycle. All experimental animal procedures were conducted in accordance with guidelines established by the Animal Committee of Xi’an Jiaotong University Health Science Center. All possible efforts were expended to minimize the number of animals used and their suffering. Three/four mice were housed together and had free access to food and water for the duration of the experiments.

### 4.2. Construction of a Mouse Model of TBI

Mice were randomly divided into 4 groups, viz. the sham group (*n* = 15), TBI group (*n* = 15), sham + VWR group (*n* = 12), and TBI + VWR group (*n* = 12). Feeney’s freefall epidural impact method was used to generate the moderate TBI model. Briefly, the mice were anesthetized with 0.75% pentobarbital sodium (10 mg/kg) via intraperitoneal injection and placed within a stereotaxic frame. The surgical area was cleaned by betadine surgical scrub following hair removal. A craniotomy of 4 mm diameter was performed at a point that was centered 1 mm posterior and 2 mm lateral to the right of the bregma. The skull cap was removed carefully without damaging the leptomeninges. Thereafter, an 80-g hammer was dropped from a height of 25 cm to induce craniocerebral injury. The exposed dura was covered with bone wax, the scalp was sutured, and then erythromycin ointment was smeared on the affected area at the end of the procedure. All mice were placed on a heating pad to maintain a core body temperature of 36–37 °C to recover after the induction of TBI. The mice in the control group underwent the same surgical procedure but without TBI (i.e., the sham-treated mice).

### 4.3. Voluntary Exercise Strategy

VWR was employed as a voluntary physical activity. Two days before TBI, twenty-four mice in the VWR group (sham + VWR group, *n* = 12; TBI + VWR group, *n* = 12) were placed in a clean polycarbonate cage with a stainless steel running wheel (12.7 cm diameter, Lafayette Instrument Company, Louisiana, USA) individually, to facilitate adaptation to the environment. VWR was initiated on the 2nd day after TBI (Figure 1A). Running activity was monitored daily for one week. The running distance and the total number of wheel rotations were measured using the Scurry VitalView Acquisition and Analysis Software (version 1.1). The mice in the other two groups were housed in standard cages without a running wheel.

### 4.4. NSS Test

The motor, sensory, and reflex activities of the mice (*n* = 12 in each group) after TBI and their neurofunctional recovery with exercise intervention were assessed using the NSS test, as described previously [52]. The test used a double-blind evaluation and was carried out two days after TBI and seven days after VWR, respectively. The NSS scale ranges from 0 to 10 (Table 1), and 1 point is awarded for lack of reflex or inability to perform a task. A maximum of 10 points indicated the most severe neurological dysfunction with failure for all tasks.

### 4.5. Motor Coordination and Balance Test

Motor coordination and balance were assessed using the beam walking assay. It was carried out once daily, started one day before the operation and lasted until nine days after TBI. The blinded evaluation was performed to limit observer bias during the whole process. Before TBI, all mice (*n* = 12 in each group) were pre-trained until they gained proficiency to walk across a wooden beam without pausing. The apparatus for the beam walking test consists of a flat bench (70 cm × 35 cm) with a wooden beam (100 cm long and 1 cm wide) located 20 cm above the bench and held in place by two posts. Each mouse was placed in the same starting position. The experimenter observed the site where the mice fell off and recorded the distance between the starting and falling positions.

### 4.6. Behavioral Tests for Anxiety

The mice (*n* = 12 in each group) were subjected to a series of behavioral tests after one week of VWR. All tests were performed according to the standard procedure and optimized for our laboratory [53]. All data were analyzed in a double-blinded manner using automated video tracking software (Smart 3.0 video tracking system, Harvard Apparatus, Boston, MA, USA). Anxiety behavior was evaluated using the OFT and EPM tests. Before the test, each mouse was placed in the environment for acclimation and baseline measurements.

#### 4.6.1. OFT

On the experiment day, the mouse was placed individually at the center of the apparatus (50 cm × 50 cm) to permit exploration for 5 min. The animals’ movements were recorded by an overhead video camcorder. The time spent in the software-defined 25 cm × 25 cm central region of the apparatus was recorded for each animal. The time spent in the center zone was measured, and the total distance traveled was calculated.

#### 4.6.2. EPM Test

The EPM apparatus consists of two enclosed arms and two open arms (35 cm in length × 6 cm in width × 15 cm in height; Harvard Apparatus, Boston, MA, USA) at an angle of 90° to each other with all arms platforms elevated 50 cm from the floor. At the start of a trial, the mouse was placed at the center with its nose directed toward the same closed arm and allowed to explore the maze freely for 5 min. The total time spent, total distance covered, distance traveled, and entries into each arm and the center were recorded digitally. The number of entries and duration of time spent in the open arms was calculated, represented as a percentage of the total number of entries (sum of the entries into the open and closed arms) and total time (sum of the duration spent in both arms). The anxiolytic effect was represented by a decrease in the percentage of the number of entries and the time spent in the open arms [54].

### 4.7. Histology and Immunofluorescence Staining

Each mouse underwent transcranial perfusion with 0.01 M phosphate-buffered saline (PBS), followed by 4% paraformaldehyde (PFA) in 0.1 M PBS (pH: 7.4), after anesthetization at 1.5 h after TBI (*n* = 3 in sham and TBI groups) or the behavioral tests (*n* = 3 in each group). The whole brain was dissected quickly and post-fixed in PFA for 24 h at 4°C. After gradient dehydration with shaking overnight, the brain tissues were embedded in optimal cutting temperature compound and sectioned coronally into slices (10-μm thick) using a cryostat microtome (CM1950; Leica Microsystems, Wetzlar, Germany). Hematoxylin and eosin staining was performed, as per the standard procedures, to assess the degree of brain tissue damage. For immunofluorescence staining, the brain sections were first treated with antigen retrieval solution (C1035, Solarbio, Beijing, China) for 15 min, washed with 0.01 M PBS for 5 min, and subsequently blocked with 5% bovine serum albumin in 0.3% Triton X-100 for 2 h at room temperature (RT). The sections were then incubated overnight at 4 °C with primary antibodies, including rabbit polyclonal anti- Iba1 (019-19741, 1:500, Wako, Japan), rat monoclonal anti-CD68 (ab53444, 1:100, Abcam, Cambridge, MA, USA), mouse monoclonal anti-NLRP3 (AG-20B-0014, 1:50, Whatman, UK), and rabbit polyclonal anti-c-fos (26192-1-AP, 1:50, Proteintech, Wuhan, China). On the following day, the sections were incubated at RT in the dark for 2 h with secondary antibodies, including Alexa Fluor 488 and 594 conjugated donkey anti-rabbit/donkey anti-mouse IgG (1:600, Invitrogen, Waltham, MA, USA) and Alexa Fluor 488 conjugated goat anti-rat IgG (1:300, Abcam). After thorough washing, the brain sections were incubated with 4′,6-diamidino-2-phenylindole (DAPI) for 5 min to label the cell nuclei and mounted with Antifade Mounting Medium (Sigma, Livonia, MI, USA). All immunofluorescence images were viewed and photographed under a fluorescent microscope (BX57, Olympus Corporation, Tokyo, Japan). The positive staining cells were counted with the Image J software (US NIH), and the double positive cells were visualized to see the colocalization. Cell density was calculated by counting the number of positive cells out of total cells (DAPI positive) in a unit area, as previously reported [55,56].

### 4.8. Western Blotting Assay

The molecules associated with the inflammatory cell signaling pathway were detected using the Western blot assay. All mice (*n* = 4 in each group) were sacrificed after anesthetization within 1.5 h after the behavioral tests. The mice perilesional cortex tissues were cut into pieces and lysed with radioimmunoprecipitation assay (RIPA) buffer (PE01, Pioneer, Shanghai, China) supplemented with a proteinase inhibitor cocktail (Roche, Mannheim, Germany) for 1 h on ice. Subsequently, lysates were harvested and centrifuged at 12,000 rpm for 15 min at 4 °C. The extracted total protein concentration was detected by a BCA protein assay kit (P0009, Beyotime Biotechnology, Shanghai, China). After that, the protein was separated on 10% sodium dodecyl-sulfate polyacrylamide gel electrophoresis gel and transferred to a polyvinylidene fluoride membrane (IPVH00010, Millipore, Billerica, MA, USA). Skim milk in 5% (*m*/*v*) (232100, BD Biosciences, San Jose, CA, USA) was used to block for 3 h at RT, then the membranes were incubated with primary antibodies overnight at 4 °C. The following antibodies were utilized after dilution in skim milk at a concentration of 1:1000: rabbit polyclonal anti-caspase-1 (ab179515, 1:1000, Abcam), rabbit polyclonal anti- interleukin (IL)-1β (ab283818, 1:1000, Abcam), rabbit polyclonal anti-IL-18 (ab207323, 1:1000, Abcam), and mouse monoclonal anti-glyceraldehyde-3-phosphate dehydrogenase (GAPDH) (60004-1-Ig, 1:10,000, Proteintech, Wuhan, China). On the following day, the membranes were washed thrice with Tris-buffered saline-Tween 20 (TBST) buffer and then incubated with horseradish peroxidase-conjugated goat anti-rabbit or goat anti-mouse IgG (1:10000, Proteintech, Wuhan, China) for 2 h at RT. Subsequently, the membranes were washed with TBST and visualized using an enhanced chemiluminescence kit (WBKLS0100, Millipore, Burlington, MA, USA). The images were scanned digitally, and the bands were quantified with densitometry using the Image J software (US NIH).

### 4.9. qRT-PCR Analysis

qRT-PCR was used to analyze the relative mRNA expression of the genes associated with the pro- or anti-inflammatory factors and NLRP3. The total RNA of the mice perilesional cortex (*n* = 5 in each group) was extracted using the TRIzol reagent (Takara, Dalian, China) and transcribed into cDNA using a reverse transcription kit (K1622, Thermo Scientific, Waltham, MA, USA). Thereafter, qPCR was performed using SYBR green dye with gene-specific primer sets and an iQ5 PCR thermal cycler (Bio-Rad, Hercules, CA, USA) with the following cycle parameters: 95 °C for 3 min, 40 cycles at 95 °C for 10 s, 60 °C for 30 s, and 72 °C for 30 s. The primer sequences are listed in Table 2. The mRNA expression of the target genes was normalized to GAPDH and quantified using the 2^−ΔΔ^ CT method.

### 4.10. Data Collection and Statistical Analysis

Data were expressed as the mean  ±  standard error of the mean. All data were analyzed using Prism 8 (GraphPad Software 8.0, Inc., San Diego, CA, USA). *p*-values ≤ 0.05 were considered statistically significant. At least three samples were used for Histology and immunofluorescence staining; four sections from each brain and three randomly selected fields were imaged and counted. Data from four samples were used for the Western blot assay, and five samples were used for the qRT-PCR assay. The two-tailed unpaired Student’s t-test was used to compare the NSS and total running wheel revolutions of the TBI and sham groups. Data on the daily running wheel revolutions were analyzed using the two-way ANOVA with VWR treatment and time. Data gathered from the other experiments, including anxiety behavior, neuroinflammatory reaction, and mRNA expression, were analyzed using the one-way ANOVA, followed by Sidak’s multiple comparisons tests. The relationship between NLRP3 inflammasome activation and anxiety-like behavior was analyzed using the two-tailed Mann-Whitney U test.

## 5. Conclusions

Our data demonstrated that VWR, which was performed at the early stage after TBI, could efficiently improve neurofunctional recovery and inhibit trauma-induced neuroinflammation, showed by the elevation in anti-inflammatory signaling and the reduction in NLRP3 activation. This further confirmed that in-time voluntary exercise could be a highly useful therapeutic strategy for neuropsychiatric disorders following TBI by targeting neuroinflammation.

## Figures and Tables

**Table 1:** Neurological Severity Score.

Task	Points
Exit circle	1
Monoparesis/hemiparesis	1
Straight walk	1
Startle reflex	1
Seeking behavior	1
Beam balancing	1
Round stick balancing	1
Beam walk: 3 cm	1
Beam walk: 2 cm	1
Beam walk: 1 cm	1
Maximal score	10

**Table 2:** Summary of Primer Sequences for Real-Time Polymerase Chain Reaction.

Gene	Forward Primer	Reverse Primer
IL-12	GTCCTCAGAAGCTAACCATCTCC	CCAGAGCCTATGACTCCATGTC
INF-γ	GAACTGGCAAAAGGATGGTGA	TGTGGGTTGTTGACCTCAAAC
CCL2	TTAAAAACCTGGATCGGAACCAA	GCATTAGCTTCAGATTTACGGGT
IL-10	GGCAGAGAAGCATGGCCCAGAA	AATCGATGACAGCGCCTCAGCC
TGF-β	CTCCCGTGGCTTCTAGTGC	GCCTTAGTTTGGACAGGATCTG
iNOS	CACCAAGCTGAACTTGAGCG	CGTGGCTTTGGGCTCCTC
CD16	TTTGGACACCCAGATGTTTCAG	GTCTTCCTTGAGCACCTGGATC
IL-1β	TGGGAAACAACAGTGGTCAGG	CCATCAGAGGCAAGGAGGAA
Ym-1	CAAGTTGAAGGCTCAGTGGCTC	CAAATCATTGTGTAAAGCTCCTCTC
Arg-1	CCAGAAGAATGGAAGAGTCAGTGT	GCAGATATGCAGGGAGTCACC
CD206	TCTTTGCCTTTCCCAGTCTCC	TGACACCCAGCGGAATTTC
NLRP3	ATTACCCGCCCGAGAAAGG	CATGAGTGTGGCTAGATCCAAG
GAPDH	GCCAAGGCTGTGGGCAAGGT	TCTCCAGGCGGCACGTCAGA

IL: interleukin, TGF: transforming growth factor, CCL: chemokine ligand, GAPGH: glyceraldehyde-3-phosphate dehydrogenase, NLRP3: NOD-like receptor thermal protein domain associated protein 3, INF: interferon.

## Data Availability

Not applicable.

## Supplementary Materials

The following supporting information can be downloaded at: https://www.mdpi.com/article/10.3390/ijms24076365/s1.

## References

[1] Liu B. (2015). Current status and development of traumatic brain injury treatments in China. Chin. J. Traumatol..

[2] Gardner A.J., Zafonte R. (2016). Neuroepidemiology of traumatic brain injury. Handb. Clin. Neurol..

[3] GBD 2016 Neurology Collaborators (2019). Global, regional, and national burden of traumatic brain injury and spinal cord in-jury, 1990–2016: A systematic analysis for the Global Burden of Disease Study 2016. Lancet Neurol..

[4] Jiang J.Y., Gao G.Y., Feng J.F., Mao Q., Chen L.G., Yang X.F., Liu J.F., Wang Y.H., Qiu B.H., Huang X.J. (2019). Traumatic brain injury in China. Lancet Neurol..

[5] Ahmed S., Venigalla H., Mekala H.M., Dar S., Hassan M., Ayub S. (2017). Traumatic brain injury and neuropsychiatric complications. Indian J. Psychol. Med..

[6] Braga M.F.M., Juranek J., Eiden L.E., Li Z., Figueiredo T.H., de Araujo Furtado M., Marini A.M. (2022). GABAergic circuits of the basolateral amygdala and generation of anxiety after traumatic brain injury. Amino Acids.

[7] Osborn A.J., Mathias J.L., Fairweather-Schmidt A.K. (2016). Prevalence of anxiety following adult traumatic brain injury: A meta-analysis comparing measures, samples and postinjury intervals. Neuropsychology.

[8] Albrecht J.S., Peters M.E., Smith G.S., Rao V. (2017). Anxiety and posttraumatic stress disorder among medicare beneficiaries after traumatic brain injury. J. Head Trauma. Rehabil..

[9] Popovitz J., Mysore S.P., Adwanikar H. (2019). Long-term effects of traumatic brain injury on anxiety-like behaviors in mice: Behavioral and neural correlates. Front. Behav. Neurosci..

[10] Tucker L.B., Burke J.F., Fu A.H., McCabe J.T. (2017). Neuropsychiatric symptom modeling in male and female c57bl/6j mice after experimental traumatic brain injury. J. Neurotrauma.

[11] Scholten A.C., Haagsma J.A., Cnossen M.C., Olff M., van Beeck E.F., Polinder S. (2016). Prevalence of and risk factors for anxiety and depressive disorders after traumatic brain injury: A systematic review. J. Neurotrauma.

[12] Diaz A.P., Schwarzbold M.L., Thais M.E., Hohl A., Bertotti M.M., Schmoeller R., Nunes J.C., Prediger R., Linhares M.N., Guarnieri R. (2012). Psychiatric disorders and health-related quality of life after severe traumatic brain injury: A prospective study. J. Neurotrauma.

[13] Zheng R.Z., Lee K.Y., Qi Z.X., Wang Z., Xu Z.Y., Wu X.H., Mao Y. (2022). Neuroinflammation following traumatic brain injury: Take it seriously or not. Front. Immunol..

[14] Jassam Y.N., Izzy S., Whalen M., McGavern D.B., El Khoury J. (2017). Neuroimmunology of traumatic brain injury: Time for a paradigm shift. Neuron.

[15] Mira R.G., Lira M., Cerpa W. (2021). Traumatic brain injury: Mechanisms of glial response. Front. Physiol..

[16] Karve I.P., Taylor J.M., Crack P.J. (2016). The contribution of astrocytes and microglia to traumatic brain injury. Br. J. Pharmacol..

[17] Liu H.D., Li W., Chen Z.R., Hu Y.C., Zhang D.D., Shen W., Zhou M.L., Zhu L., Hang C.H. (2013). Expression of the NLRP3 inflammasome in cerebral cortex after traumatic brain injury in a rat model. Neurochem. Res..

[18] Irrera N., Russo M., Pallio G., Bitto A., Mannino F., Minutoli L., Altavilla D., Squadrito F. (2020). The role of NLRP3 inflammasome in the pathogenesis of traumatic brain injury. Int. J. Mol. Sci..

[19] Wei X., Hu C.C., Zhang Y.L., Yao S.L., Mao W.K. (2016). Telmisartan reduced cerebral edema by inhibiting NLRP3 inflammasome in mice with cold brain injury. J. Huazhong Univ. Sci. Technol. Med. Sci..

[20] Ma J., Xiao W., Wang J., Wu J., Ren J., Hou J., Gu J., Fan K., Yu B. (2016). Propofol inhibits NLRP3 inflammasome and attenuates blast-induced traumatic brain injury in rats. Inflammation.

[21] O’Brien W.T., Pham L., Symons G.F., Monif M., Shultz S.R., McDonald S.J. (2020). The NLRP3 inflammasome in traumatic brain injury: Potential as a biomarker and therapeutic target. J. Neuroinflamm..

[22] Rashno M., Ghaderi S., Nesari A., Khorsandi L., Farbood Y., Sarkaki A. (2020). Chrysin attenuates traumatic brain injury-induced recognition memory decline, and anxiety/depression-like behaviors in rats: Insights into underlying mechanisms. Psychopharmacology.

[23] Zhao J., Qu D., Xi Z., Huan Y., Zhang K., Yu C., Yang D., Kang J., Lin W., Wu S. (2021). Mitochondria transplantation protects traumatic brain injury via promoting neuronal survival and astrocytic BDNF. Transl. Res..

[24] Mele C., Pingue V., Caputo M., Zavattaro M., Pagano L., Prodam F., Nardone A., Aimaretti G., Marzullo P. (2021). Neuroinflammation and hypothalamo-pituitary dysfunction: Focus of traumatic brain injury. Int. J. Mol. Sci..

[25] Rodgers K.M., Bercum F.M., McCallum D.L., Rudy J.W., Frey L.C., Johnson K.W., Watkins L.R., Barth D.S. (2012). Acute neuroimmune modulation attenuates the development of anxiety-like freezing behavior in an animal model of traumatic brain injury. J. Neurotrauma.

[26] Rodgers K.M., Deming Y.K., Bercum F.M., Chumachenko S.Y., Wieseler J.L., Johnson K.W., Watkins L.R., Barth D.S. (2014). Reversal of established traumatic brain injury-induced, anxiety-like behavior in rats after delayed, post-injury neuroimmune suppression. J. Neurotrauma.

[27] Yang F., Wang Z., Wei X., Han H., Meng X., Zhang Y., Shi W., Li F., Xin T., Pang Q. (2014). NLRP3 deficiency ameliorates neurovascular damage in experimental ischemic stroke. J. Cereb. Blood Flow. Metab..

[28] Irrera N., Pizzino G., Calo M., Pallio G., Mannino F., Fama F., Arcoraci V., Fodale V., David A., Francesca C. (2017). Lack of the Nlrp3 inflammasome improves mice recovery following traumatic brain injury. Front. Pharmacol..

[29] Zheng B., Zhang S., Ying Y., Guo X., Li H., Xu L., Ruan X. (2018). Administration of dexmedetomidine inhibited NLRP3 inflammasome and microglial cell activities in hippocampus of traumatic brain injury rats. Biosci. Rep..

[30] Kuwar R., Rolfe A., Di L., Xu H., He L., Jiang Y., Zhang S., Sun D. (2019). A novel small molecular NLRP3 inflammasome inhibitor alleviates neuroinflammatory response following traumatic brain injury. J. Neuroinflamm..

[31] Ismael S., Nasoohi S., Ishrat T. (2018). MCC950, the selective inhibitor of nucleotide oligomerization domain-like receptor protein-3 inflammasome, protects mice against traumatic brain injury. J. Neurotrauma.

[32] Chen C., Nakagawa S., An Y., Ito K., Kitaichi Y., Kusumi I. (2017). The exercise-glucocorticoid paradox: How exercise is beneficial to cognition, mood, and the brain while increasing glucocorticoid levels. Front. Neuroendocrinol..

[33] Xiong Y., Mahmood A., Chopp M. (2009). Emerging treatments for traumatic brain injury. Expert Opin. Emerg. Drugs.

[34] Stephan J.S., Sleiman S.F. (2019). Exercise factors as potential mediators of cognitive rehabilitation following traumatic brain injury. Curr. Opin. Neurol..

[35] Kodali M., Mishra V., Hattiangady B., Attaluri S., Gonzalez J.J., Shuai B., Shetty A.K. (2021). Moderate, intermittent voluntary exercise in a model of Gulf War Illness improves cognitive and mood function with alleviation of activated microglia and astrocytes, and enhanced neurogenesis in the hippocampus. Brain Behav. Immun..

[36] Griesbach G.S., Tio D.L., Nair S., Hovda D.A. (2014). Recovery of stress response coincides with responsiveness to voluntary exercise after traumatic brain injury. J. Neurotrauma.

[37] Amorós-Aguilar L., Portell-Cortés I., Costa-Miserachs D., Torras-Garcia M., Riubugent-Camps È., Almolda B., Coll-Andreu M. (2020). The benefits of voluntary physical exercise after traumatic brain injury on rat’s object recognition memory: A comparison of different temporal schedules. Exp. Neurol..

[38] Griesbach G.S., Tio D.L., Vincelli J., McArthur D.L., Taylor A.N. (2012). Differential effects of voluntary and forced exercise on stress responses after traumatic brain injury. J. Neurotrauma.

[39] Scrimgeour A.G., Condlin M.L., Loban A., DeMar J.C. (2021). Omega-3 fatty acids and vitamin d decrease plasma t-tau, gfap, and uch-l1 in experimental traumatic brain injury. Front. Nutr..

[40] Zhao Z., Sabirzhanov B., Wu J., Faden A.I., Stoica B.A. (2015). Voluntary exercise preconditioning activates multiple antiapoptotic mechanisms and improves neurological recovery after experimental traumatic brain injury. J. Neurotrauma.

[41] Martínez-Drudis L., Amorós-Aguilar L., Torras-Garcia M., Serra-Elias B., Costa-Miserachs D., Portell-Cortés I., Coll-Andreu M. (2021). Delayed voluntary physical exercise restores “when” and “where” object recognition memory after traumatic brain injury. Behav. Brain Res..

[42] Risbrough V.B., Vaughn M.N., Friend S.F. (2022). Role of inflammation in traumatic brain injury-associated risk for neuropsychiatric disorders: State of the evidence and where do we go from here. Biol. Psychiatry.

[43] Osborn A.J., Mathias J.L., Fairweather-Schmidt A.K., Anstey K.J. (2017). Anxiety and comorbid depression following traumatic brain injury in a community-based sample of young, middle-aged and older adults. J. Affect. Disord..

[44] Yurgil K.A., Barkauskas D.A., Vasterling J.J., Nievergelt C.M., Larson G.E., Schork N.J., Litz B.T., Nash W.P., Baker D.G. (2014). Association between traumatic brain injury and risk of posttraumatic stress disorder in active-duty Marines. JAMA Psychiatry.

[45] Stein M.B., Kessler R.C., Heeringa S.G., Jain S., Campbell-Sills L., Colpe L.J., Fullerton C.S., Nock M.K., Sampson N.A., Schoenbaum M. (2015). Prospective longitudinal evaluation of the effect of deployment-acquired traumatic brain injury on posttraumatic stress and related disorders: Results from the Army Study to Assess Risk and Resilience in Servicemembers (Army STARRS). Am. J. Psychiatry.

[46] Rodney T., Taylor P., Dunbar K., Perrin N., Lai C., Roy M., Gill J. (2020). High IL-6 in military personnel relates to multiple traumatic brain injuries and post-traumatic stress disorder. Behav. Brain Res..

[47] Stein M.B., Jain S., Giacino J.T., Levin H., Dikmen S., Nelson L.D., Vassar M.J., Okonkwo D.O., Diaz-Arrastia R., Robertson C.S. (2019). Risk of posttraumatic stress disorder and major depression in civilian patients after mild traumatic brain injury: A track-tbi study. JAMA Psychiatry.

[48] Devoto C., Arcurio L., Fetta J., Ley M., Rodney T., Kanefsky R., Gill J. (2017). Inflammation relates to chronic behavioral and neurological symptoms in military personnel with traumatic brain injuries. Cell Transplant..

[49] Chiu C.C., Liao Y.E., Yang L.Y., Wang J.Y., Tweedie D., Karnati H.K., Greig N.H., Wang J.Y. (2016). Neuroinflammation in animal models of traumatic brain injury. J. Neurosci. Methods.

[50] Simon D.W., McGeachy M.J., Bayır H., Clark R.S., Loane D.J., Kochanek P.M. (2017). The far-reaching scope of neuroinflammation after traumatic brain injury. Nat. Rev. Neurol..

[51] Semple B.D., Bye N., Rancan M., Ziebell J.M., Morganti-Kossmann M.C. (2010). Role of CCL2 (MCP-1) in traumatic brain injury (TBI): Evidence from severe TBI patients and CCL2−/− mice. J. Cereb. Blood Flow. Metab..

[52] Flierl M.A., Stahel P.F., Beauchamp K.M., Morgan S.J., Smith W.R., Shohami E. (2009). Mouse closed head injury model induced by a weight-drop device. Nat. Protoc..

[53] Ge Q., Hu X., Ma N., Sun M., Zhang L., Cai Z., Tan R., Lu H. (2021). Maternal high-salt diet during pregnancy impairs synaptic plasticity and memory in offspring. Faseb J..

[54] Chen W.J., Niu J.Q., Chen Y.T., Deng W.J., Xu Y.Y., Liu J., Luo W.F., Liu T. (2021). Unilateral facial injection of botulinum neurotoxin a attenuates bilateral trigeminal neuropathic pain and anxiety-like behaviors through inhibition of TLR2-mediated neuroinflammation in mice. J. Headache Pain.

[55] Verdonk F., Roux P., Flamant P., Fiette L., Bozza F.A., Simard S., Lemaire M., Plaud B., Shorte S.L., Sharshar T. (2016). Phenotypic clustering: A novel method for microglial morphology analysis. J. Neuroinflamm..

[56] Zhuang H., Yang J., Huang Z., Liu H., Li X., Zhang H., Wang J., Yu S., Liu K., Liu R. (2020). Accelerated age-related decline in hippocampal neurogenesis in mice with noise-induced hearing loss is associated with hippocampal microglial degeneration. Aging.

