# Supplementary material for: Voluntary Exercise to Reduce Anxiety Behaviour in Traumatic Brain Injury Shown to Alleviate Inflammatory Brain Response in Mice
Source: Int J Mol Sci. 2023 Mar 28;24(7):6365. doi: 10.3390/ijms24076365 (PMC10093932; doi:10.3390/ijms24076365)
Supplement: Supplementary file 1 [file ijms-24-06365-s001.zip › ijms-2287508-supplementary.pdf]

Voluntary Exercise-Induced Reduction of Inflammatory Response is Involved in Anxiety Behavior Alleviation in Traumatic Brain Injury Mice

Supplementary Materials

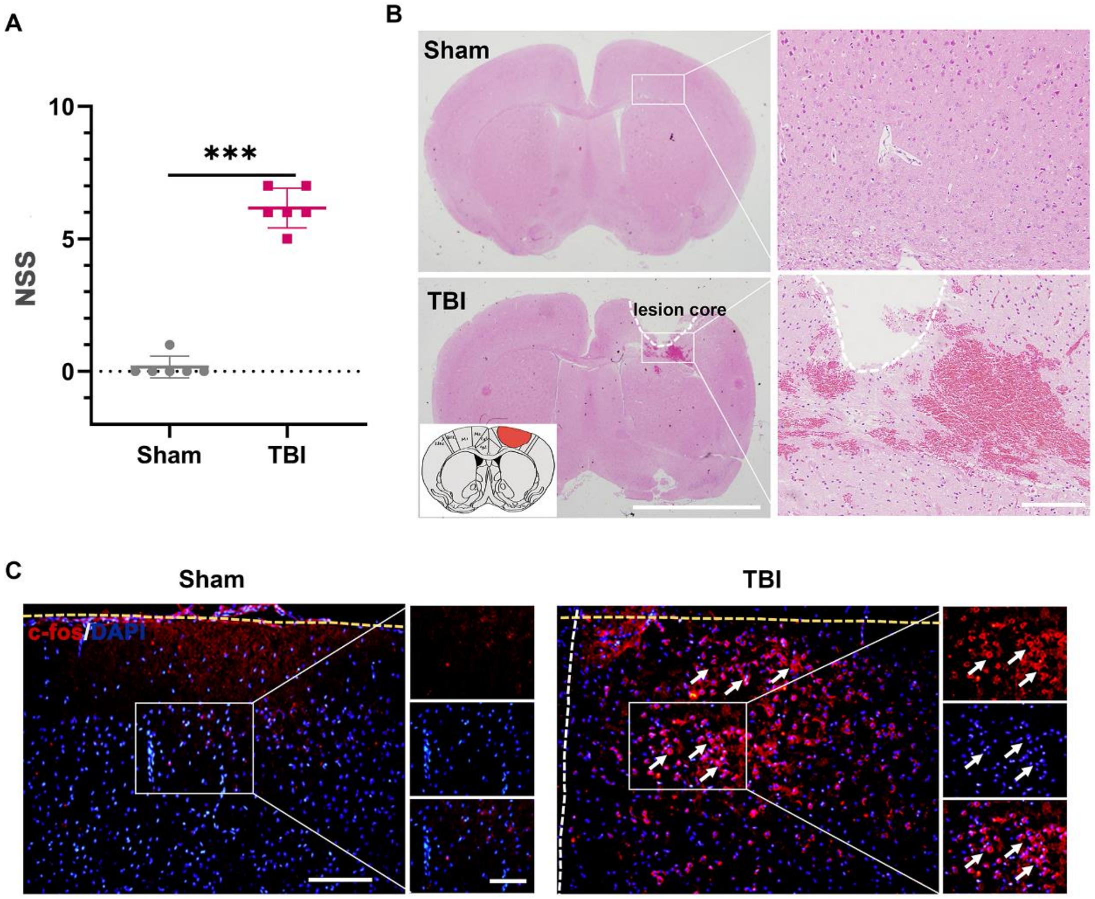

Figure S1. Damage to neurological function and brain tissue. (**A**) Neurological dysfunction, showed by NSS, was observed at 2 days after TBI, n = 6. (**B**) Obvious tissue loss was observed in cerebral cortex, n = 3. (**C**) The number of c-fos positive damaged neurons detected at 1.5 hours post-TBI in perilesional cortex increased significantly, n = 3. ***$p < 0.0001$ vs. Sham. Unpaired student's t test. scale bar =100 μm. NSS, neurological severity score; TBI, traumatic brain injury; H&E, hematoxylin-eosin.

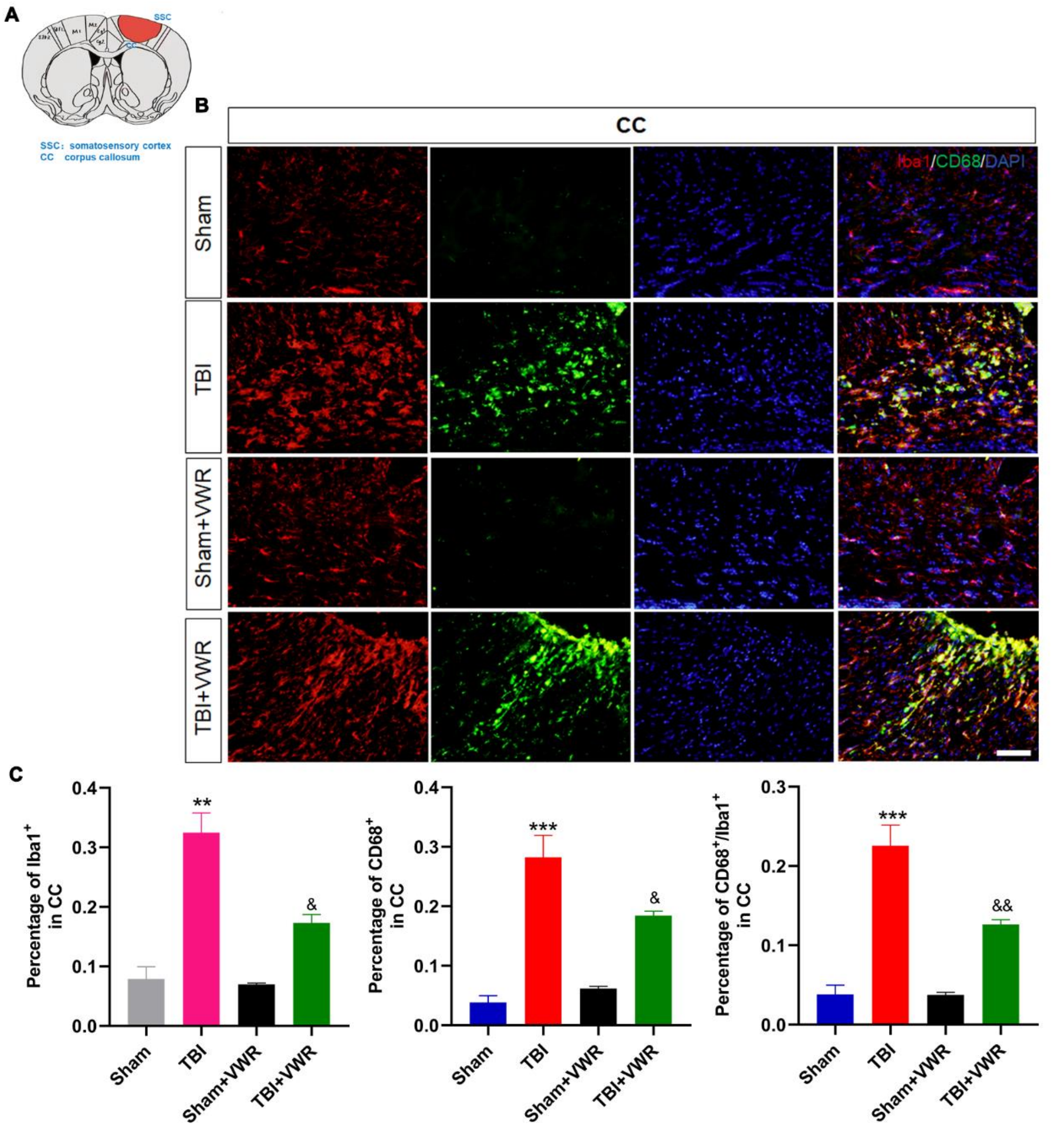

Figure S2. Activation of microglia after TBI in the CC. (**A**) Illustration of structural divisions in cerebral cortex. (**B-C**)The number of Iba1$^+$ cells, CD68$^+$ cells and Iba1$^+$/CD68$^+$ cells increased significantly after TBI and decreased after VWR. $^{***}p < 0.0001$, $^{**}p < 0.01$ vs. Sham, $^{\&\&}p < 0.01$, $^{\&}p < 0.05$ vs. TBI. One-way ANOVA, Post-hoc Sidak's multiple comparisons test; n =3. Iba1: red; CD68: green; DAPI: blue; scale bar =100 μm. CC, corpus callosum; VWR, voluntary wheel running; TBI, traumatic brain injury.

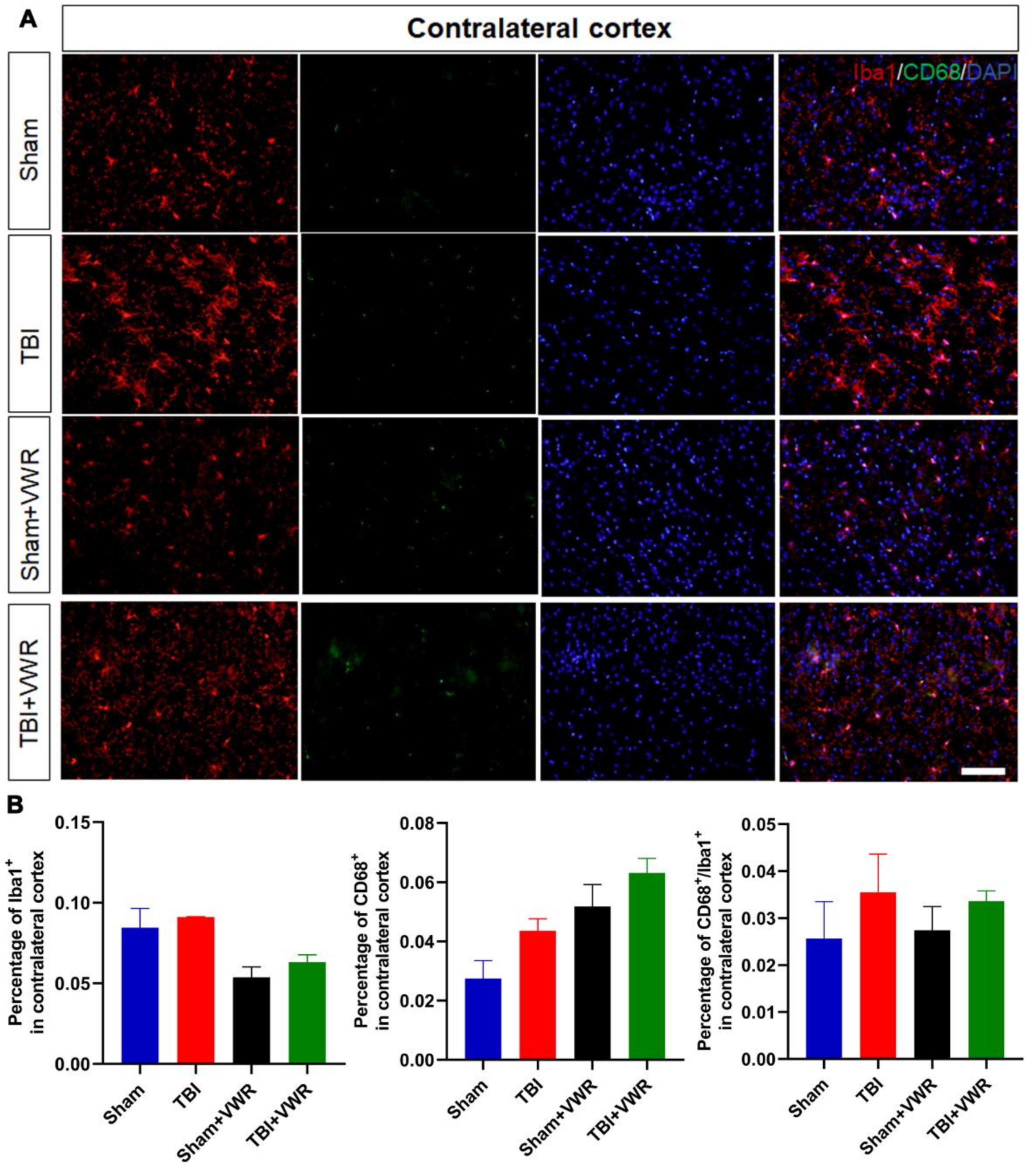

Figure S3. Activation of microglia after TBI in the contralateral cortex. (**A-B**) No significant changes was found regarding the number of Iba1+, CD68+ and Iba1+/CD68+ cells between different groups in the contralateral cerebral cortex. One-way ANOVA, Post-hoc Sidak's multiple comparisons test; n =3. Iba1: red; CD68: green; DAPI: blue; scale bar =100 μm. VWR, voluntary wheel running; TBI, traumatic brain injury.
